# Identification and characterization of histone deacetylases in tomato (*Solanum lycopersicum*)

**DOI:** 10.3389/fpls.2014.00760

**Published:** 2015-01-06

**Authors:** Linmao Zhao, Jingxia Lu, Jianxia Zhang, Pei-Ying Wu, Songguang Yang, Keqiang Wu

**Affiliations:** ^1^Key Laboratory of Plant Resources Conservation and Sustainable Utilization, South China Botanical Garden, Chinese Academy of SciencesGuangzhou, China; ^2^College of Life Sciences, University of Chinese Academy of SciencesBeijing, China; ^3^Institute of Plant Biology, National Taiwan UniversityTaipei, Taiwan

**Keywords:** histone deacetylases, subcellular localization, gene expression, MADS-box proteins, tomato

## Abstract

Histone acetylation and deacetylation at the N-terminus of histone tails play crucial roles in the regulation of eukaryotic gene activity. Histone acetylation and deacetylation are catalyzed by histone acetyltransferases and histone deacetylases (HDACs), respectively. A growing number of studies have demonstrated the importance of histone deacetylation/acetylation on genome stability, transcriptional regulation, development and response to stress in *Arabidopsis*. However, the biological functions of HDACs in tomato have not been investigated previously. Fifteen HDACs identified from tomato (*Solanum lycopersicum*) can be grouped into RPD3/HDA1, SIR2 and HD2 families based on phylogenetic analysis. Meanwhile, 10 members of the RPD3/HDA1 family can be further subdivided into four groups, namely Class I, Class II, Class III, and Class IV. High similarities of protein sequences and conserved domains were identified among SlHDACs and their homologs in *Arabidopsis*. Most SlHDACs were expressed in all tissues examined with different transcript abundance. Transient expression in *Arabidopsis* protoplasts showed that SlHDA8, SlHDA1, SlHDA5, SlSRT1 and members of the HD2 family were localized to the nucleus, whereas SlHDA3 and SlHDA4 were localized in both the cytoplasm and nucleus. The difference in the expression patterns and subcellular localization of SlHDACs suggest that they may play distinct functions in tomato. Furthermore, we found that three members of the RPD3/HDA1 family, SlHDA1, SIHDA3 and SlHDA4, interacted with TAG1 (TOMATO AGAMOUS1) and TM29 (TOMATO MADS BOX29), two MADS-box proteins associated with tomato reproductive development, indicating that these HDACs may be involved in gene regulation in reproductive development.

## Introduction

In eukaryote cells, genetic information encoded by DNA is packed extensively forming the chromatin structure. The fundamental unit of chromatin is a nucleosome containing a histone octamer (two copies of histone H2A, H2B, H3, and H4) wrapped on approximately 147 base pairs of DNA (Luger et al., 1997). Each histone has a structured globular domain and an unstructured amino-terminal tail that extends from the core nucleosome (Campos and Reinberg, 2009). The N-terminal tails of histone proteins provide sites for a variety of post-translational modifications, such as acetylation, phosphorylation, methylation, glycosylation, ubiquitation and ADP-ribosylation [3]. Among these modifications, histone acetylation is one of the well characterized post-translational modifications (Allfrey et al., 1964; Lusser et al., 2001).

The acetylation state of the ε-amino group of conserved lysine residues within all four core histones was regulated by the opposing activities of histone acetyltransferases (Brownell et al., 1996) and histone deacetylases (HDACs) (Nagy et al., 1997). HDACs can remove acetyl groups from histone and non-histone substrates including transcription factors and other proteins involved in DNA repair and replication, metabolism, cytoskeleton dynamics, apoptosis and cell signaling (Yang and Seto, 2007). Based on sequence similarity and cofactor dependency, HDACs in all eukaryotes are divided into three families: RPD3/HDA1 (Reduced Potassium Dependence 3/Histone Deacetylase 1), SIR2 (Silent Information Regulator 2), and plant-specific HD2 (Histone Deacetylase 2) (Pandey et al., 2002; Yang and Seto, 2007). Members of the SIR2 family (sirtuins) have a catalytic domain that is characterized by the requirement for nicotine adenine dinucleotide (NAD) as a cofactor (Haigis and Guarente, 2006), while members of the RPD3/HDA1 family share sequence homology in the HDAC domain and require the Zn^2+^ cofactor for deacetylase activity (Yang and Seto, 2007).

In the past decade, plant HDACs have drawn considerable research attention and an increasing number of HDACs were purified, identified and characterized from plants such as maize, *Arabidopsis*, rice, barley (Demetriou et al., 2009), potato (Lagace et al., 2003), grape (Busconi et al., 2009), tobacco (Bourque et al., 2011), and tomato (Cigliano et al., 2013b). The *Arabidopsis* genome encodes 18 HDACs. Twelve of them belong to the RPD3/HDA1 superfamily, two are the SIR2 family and four are members of the plant specific HD2 family (Pandey et al., 2002; Alinsug et al., 2009). The *Arabidopsis* HDACs play a vital role in regulating gene expression in various biological processes. For instance, *HDA6* was involved in transgene silencing and maintain of DNA methylation (Aufsatz et al., 2002; Probst et al., 2004). While *HDA19*, the closest homolog of *HDA6*, was shown to be important for proper vegetative development as *hda19* mutants displayed various developmental abnormalities (Tian and Chen, 2001; Long et al., 2006; Zhou et al., 2013). *HDA18* is required for the cellular patterning in the root epidermis (Xu et al., 2005; Liu et al., 2013a), whereas *HDA7* is crucial for female gametophyte development and embryogenesis in *Arabidopsis* (Cigliano et al., 2013a). Furthermore, HDA14 is an α-tubulin decetylase associated with α/β-tubulin and enriched in microtubule fractions by direct association with the PPP-type phosphatases PP2A (Tran et al., 2012). Silencing of *HD2A* in *Arabidopsis* resulted in aborted seed development (Wu et al., 2000), while overexpression of *HD2A* caused morphological defects of leaves and flowers, delayed flowering and aborted seed development (Zhou et al., 2004). In addition, HD2A and HD2B were found to act independently with ASYMMETRIC LEAVES1 (AS1) and AS2 to control miR165/166 distribution and the development of adaxial–abaxial leaf polarity (Ueno et al., 2007). More recently, the SRT2 in *Arabidopsis* was showed to be predominantly localized at the inner mitochondrial membrane and to interact with a small number of protein complexes mainly involved in energy metabolism and metabolite transport (Konig et al., 2014).

The molecular mechanisms of fruit development are not well-understood. Genetic studies in *Arabidopsis* have uncovered the genetic network that patterns the *Arabidopsis* fruit. For instance, the members of MADS-box transcription factor family, FRUITFULL (FUL) and SHATTERPROOF 1/2 (SHP 1/2), are necessary for proper valve development (Gu et al., 1998; Ferrandiz et al., 2000). Furthermore, INDEHISCENT (IND) and ALCATRAZ (ALC), two bHLH transcription factors, are both necessary for the differentiation of the dehiscence zone between the valve and replum regions (Girin et al., 2011; Groszmann et al., 2011). Previous data also showed that HDACs were involved in fruit development. For instance, *AtHDA19* mutations induce embryonic defects and seed set reduction (Tian et al., 2003). Silencing and overexpression of *AtHD2A* both severely affect seed development (Wu et al., 2000; Zhou et al., 2004). In addition, *hda6* mutants were reported to exhibit reduced fertility (Aufsatz et al., 2002), while mutations of *AtHDA7* led to partial failure of ovule/female gametophyte development and seed abortion (Cigliano et al., 2013a).

Compared to *Arabidopsis*, relatively few HDACs were characterized in other plant species. In this study, 15 *HDACs* were identified in the tomato genome. The expression patterns and subcellular localization of tomato HDACs (SlHDACs) were investigated. Furthermore, the interaction between SlHDACs and the MADS-box proteins involved in reproductive development sheds light on potential functions of SlHDACs during reproductive development.

## Materials and methods

### Plant material and growth conditions

*Solanum lycopersicum* cultivar Henz1706 was kindly provided by Wang Ying (South China Botanical Garden). Plants were grown in soil in a controlled-environment greenhouse with a long photoperiod (16 h light/8 h dark) at 23 ± 1°C. The tomato seeds were accelerated germination by putting the seeds in a 28°C incubator for 2 days before sowing.

### Identification of SlHDAC genes

The HDACs sequences of *Arabidopsis thaliana* obtained in the TAIR database (http://www.arabidopsis.org/) were used to perform a search in the *Solamum lycopersicum* genome using the BLASTP program in the SGN browser (http://solgenomics.net/tools/blast/index.pl). All the sequences acquired were removed the duplicates and analyzed for the recognizable domains using BLAST-based NCBI conserved domain searches (http://www.ncbi.nlm.nih.gov/Structure/lexington/lexington.cgi). The protein sequences were further verified using the HMMER-based SMAT Website (http://smart.embl-heidelberg.de/) and the Pfam program (http://pfam.janelia.org/). The domain architecture was drawn using DOG2.0 software (Ren et al., 2009).

### Phylogenetic construction

The tomato HDAC proteins identified in this work along with the proteins from *Arabidopsis* were aligned with ClustalX. The phylogenetic analysis was performed using the MEGA3.0 program (Kumar et al., 2004). Neighbor-Joining method and the Poisson correction were used to infer the evolutionary history and compute the evolutionary distances. In addition, all positions containing alignment gaps and missing data were eliminated only in pairwise sequence comparisons.

### Quantitative real-time reverse transcription-PCR (qRT-PCR) assays

Total RNA was extracted with Trizol reagent (Invitrogen) according to the manufacturer's protocol. The cDNAs were synthesized from 2 μg total RNA using the TransScript™ One-Step gDNA Removal and cDNA Synthesis Supermix kit (TransGen Biotech). Real-Time PCR was performed with iTaq™ Universal SYBR® Green Supermix (BIO-RAD) using ABI 7500 Fast Real-Time PCR system. The gene-specific primers for real-time PCR were designed by primer 3.0 (Untergasser et al., 2012) and listed in Supplementary Table 1. Tomato *Actin* (Solyc03g078400) served as an internal control.

### Subcellular localization assays

The full length cDNAs of *SlHDACs* were subcloned into the pSAT6-EYFP_N1 vector (Tzfira et al., 2005) to create the SlHDAC-YFP constructs. The protoplast isolation and transient expression were conducted as described previously (Yoo et al., 2007). Mesophyll protoplasts were isolated using the well-expanded leaves from 3-week-old *Arabidopsis* Col-0 plants. The SlHDAC-YFP fusion plasmid and the nuclear maker VirD2-NLS mCherry were co-transfected into protoplasts (3 × 10^4^ protoplasts) using the PEG-calcium solution (0.4 g/mL PEG 4000, 0.2 M mannitol, 0.1 M CaCl_2_). After washed and resuspended with W5 solution (154 mM Nacl, 125 mM CaCl_2_, 5 mM KCl, 5 mM glucose, 2 mM MES), mesophyll protoplasts were incubated under white light for 12–18 h. The YFP fluorescence was examined and imaged with a confocal microscope.

### Yeast two-hybrid, BiFC and pull-down assays

Yeast two-hybrid assays were performed using the Matchmaker™ Gold Yeast Two-Hybrid Systems (Clontech). Constructs were generated by cloning SlHDACs into pGADT7 vectors and four selected MADS-box genes into pGBKT7 vectors. Different pairs of bait and prey constructs were co-transformed into yeast strain Gold by the lithium acetate method, and yeast cells were grown on minimal medium/-Leu-Trp according to the manufacturer's instructions. Transformed colonies were plated onto minimal medium/-Leu/-Trp/-Ade/-His and minimal medium/-Leu/-Trp/-Ade/-His plates containing 4 mg/mL X-α-Gal.

For BiFC assay, the cDNAs of SIHDA1, SIHDA2, SIHDA4, TAG1, and TM29 were cloned into serial pSAT1 vectors (Tzfira et al., 2005) containing either amino- or carboxyl terminal Enhanced Yellow Fluorescence Protein (EYFP) fragments by In-Fusion cloning (Clontech, In-Fusion® HD Cloning Kit, Cat#639650). Plasmids of the corresponding N- and C-terminal fusions of YFP were cotransformed into *Arabidopsis* protoplasts as described previously (Walter et al., 2004; Yoo et al., 2007). The protoplasts were incubated for 12–16 h, and the fluorescence was determined using a confocal microscope (ZEISS-510Meta). The YFP fluorescence was excited by a 514-nm laser and captured at 523–600 nm, and the chlorophyll autofluorescence was captured at 650–750 nm.

The procedures used for pull-down assays were described previously with some modifications (Yang et al., 2008; Liu et al., 2013b). GST, GST-SIHDA1, GST-SIHDA3, and GST-SIHDA4 recombinant proteins were incubated with 30 ul of GST resin in a binding buffer (50 mM Tris-HCl, pH 7.5, 100 mM NaCl, 0.25% Triton X-100, and 35 mM β-mercaptoethanol) for 2 h at 4°C, the binding reaction was washed three times with the binding buffer and then the TM29-His or TAG1-His recombinant protein was added and incubated for an additional 2 h at 4°C. After extensive washing, the pulled-down proteins were eluted by boiling, separated by 10% SDS-PAGE, and detected by immunoblotting using an anti-His antibody.

## Results

### Identification of HDACs in tomato

**Figure 1:**
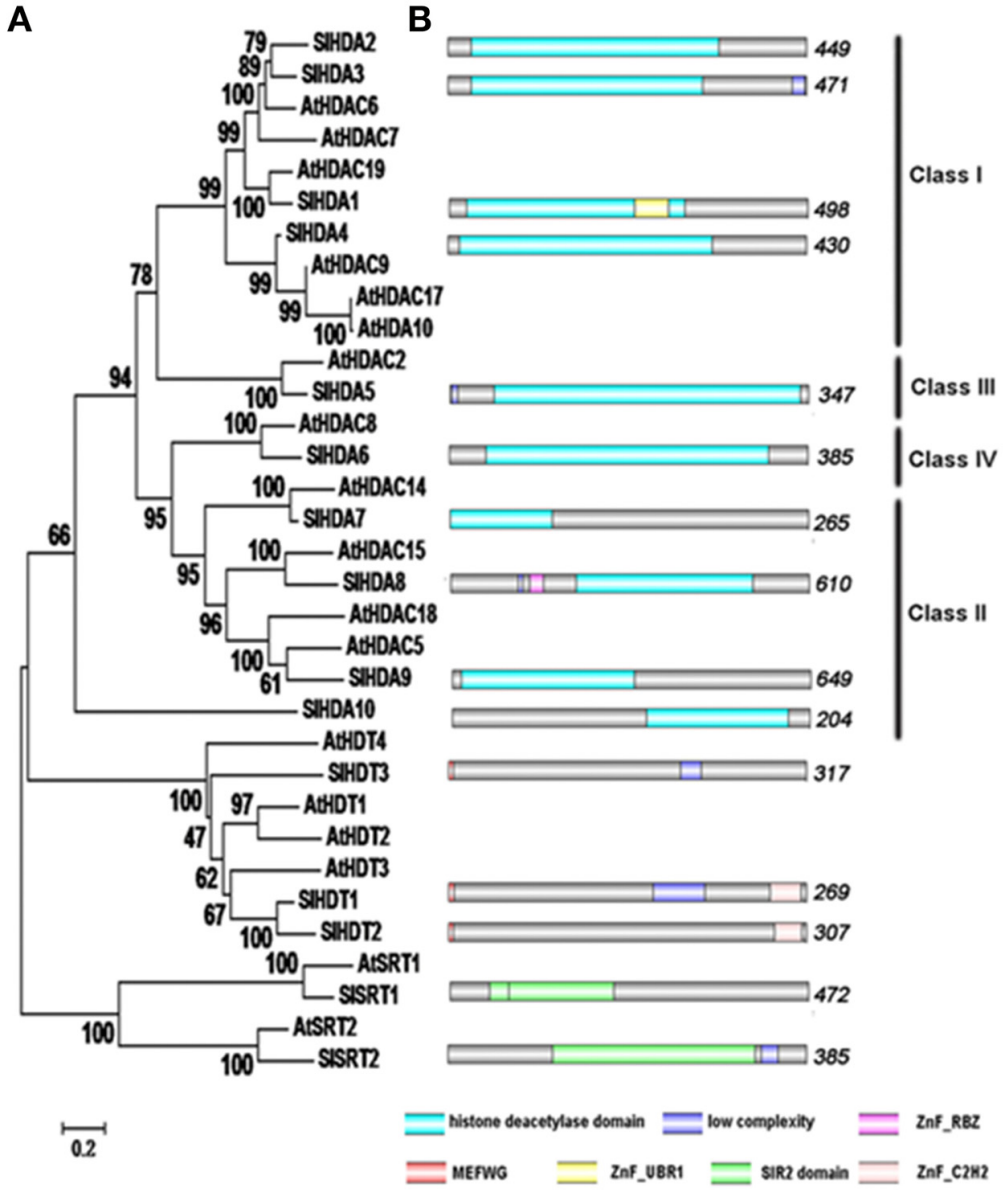
**Phylogenetic analysis and domain organization of HDACs in tomato. (A)** The neighbor joining phylogenetic tree constructed by MEGA 3 refers to the evolutionary relationship between the HDACs in tomato and *Arabidopsis*. **(B)** Domain architecture of the HDACs in tomato was drawn by DOG 2.0. The location and size of domains are shown by different color as indicated. The proteins belonging to each family are grouped together.

The HDACs of *Arabidopsis* amino acid sequences were used as queries to search against the SGN annotation database with the BLAST program. All sequences with an E-value below 10^−2^ were selected for further analysis. NCBI Conserved Domain Search, Pfam and SMART database were used to confirm each candidate protein sequence. Previously, 14 genes were identified as deduced *HDAC* genes in tomato (Cigliano et al., 2013b). We identified a new *HDAC* gene, *SlHDA10*, belonging to the *RPD3/HDA1* family in the tomato genome (Table 1, Figure 1). Fifteen *HDAC* genes distribute in different tomato chromosomes with various numbers of exons (Table 1). To further investigate the evolutionary relationships, phylogenetic analyses were performed using the Mega 3.0 software. The phylogenetic tree indicates that 15 HDACs in tomato can be divided into three clades: RPD3/HDA1, SIR2, and HD2 families (Table 1, Figure 1A). There are 10 members in the RPD3/HDA1 family, which can be further divided into four subclasses as Class I, Class II, Class III, and Class IV. In addition, there are two and three members in the SIR2 and HD2 family, respectively.

**Table 1:** **Tomato histone deacetylase proteins**.

**HDAC gene family**	**Gene name^a^**	**Loc. symbol^b^**	**Accession number^c^**	**ORF length(bp)^d^**	**protein length^e^**	**Localization^f^**	**Number of exons**
RPD3/HDA1	*SlHDA1*	Solyc09g091440	XP_004247825	1497	498	nuc	7
	*SlHDA3*	Solyc06g071680	XP_004241512	1416	471	nuc	6
	*SlHDA2*	Solyc03g112410	XP_004236540	1350	449	per	5
	*SlHDA4*	Solyc11g067020	XP_004251031	1293	430	cyto	14
	*SlHDA5*	Solyc08g065350	XP_004245106	1044	347	C	13
	*SlHDA9*	Solyc03g115150	XP_004235971	1933	649	cyto	14
	*SlHDA8*	Solyc03g119730	XP_004235741	1833	610	nuc	17
	*SlHDA7*	Solyc01g009110	XP_004228472	798	265	Plas	4
	*SlHDA6*	Solyc06g074080	XP_004241339	1148	385	cyto	3
	*SlHDA10*	Solyc01g009120	XP_004228472	615	204	C	6
SIR2	*SlSRT1*	Solyc07g065550	XP_004244044	1419	472	nuc	14
	*SlSRT2*	Solyc04g009430	XP_004236824	1158	385	M	11
HD2	*SlHDT2*	Solyc10g085560	XP_004249622	924	307	nuc	11
	*SlHDT3*	Solyc11g066840	XP_004251012	954	317	nuc	7
	*SlHDT1*	Solyc09g009030	XP_004246566	810	269	nuc	10

a *Systematic designation given to tomato histone deacetylase*.

b *Accession number of SGN (http://solgenomics.net) locus ID*.

c *Accession numbers of full-length protein sequence available at NCBI (http://www.ncbi.nlm.nih.gov/)*.

d *Length of open reading frame (number of base pair)*.

e *Length of protein (number of amino acid)*.

f *Localization of tomato histone deacetylase proteins supported by WoLF PSORT (http://www.genscript.com/psort/wolf_psort.html)*.

*Nuc, nucleus; per, peroxisome; Plas, plasma membrane; cyto, cytoplasm; M, mitochondrion; C, chloroplast*.

The conserved domains were predicted with hammer-based website Smart and Pfam (Figure 1B). The members of RPD3/HDA1 family all have a deacetylase catalytic domain. Both SlHDA1 and SlHDA8 have the zinc-finger domain which might be involved in the binding of DNA. The N-terminus of the HD2 family contains the conserved typical pentapeptide motif (MEFWG) (see Supplementary Figure 1). In addition, SlHDT2 and SlHDT1 both contain a C_2_H_2_ zinc-finger that might be involved in protein-protein interaction. Furthermore, the members of SIR2 family have the conserved domain that is dependent on NAD.

### The expression pattern of SlHDACs

**Figure 2:**
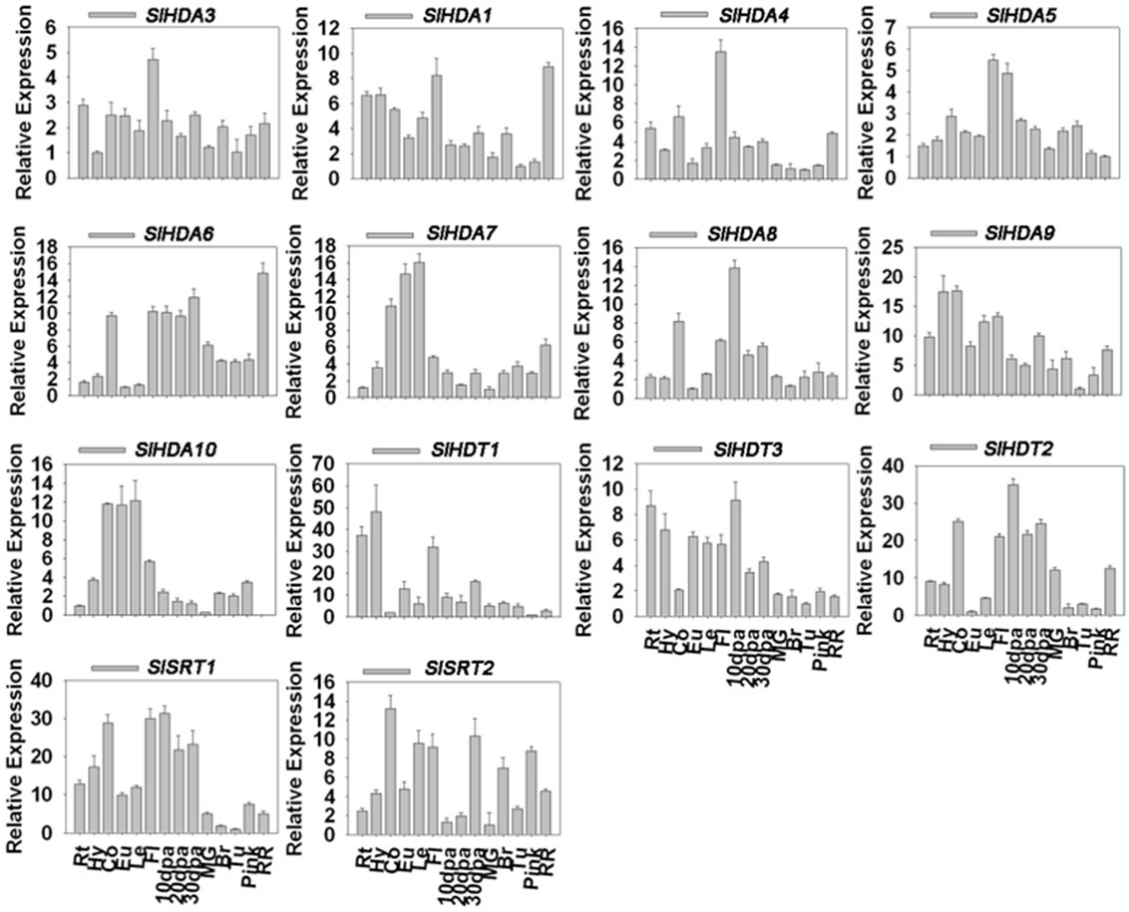
**Quantitative RT-PCR analysis of the expression of *HDACs* in different tissues and developmental stages**. Roots (Rt), hypocotyls (Hy), cotyledons (Co), euphylla (Eu), and leaves (Le) of 5-week-old plate cultured plants and flowers (Fl), fruits at 10 days post anthesis (10 dpa), 20 dpa, 30 dpa, mature green stage (MG), break stage (Br), turning stage (Tu), pink stage (pink), and red ripe stage (RR) were collected for total RNA isolation. RT-PCR was amplified using gene-specific primers. The tomato *Actin* (*Solyc03g078400*) was used as an internal control.

Considering the typical fruit development and maturation processes of tomato, we focused on the several important stages such as fruits on 10, 20, and 30 days post anthesis (dpa) as well as fruits of the maturing green stage (MG), breaker stage, turning stage, pink stage and red ripe stage (Teyssier et al., 2008) (see Supplementary Figure 2). Except for *SlHDA2*, the transcripts of other 14 *SlHDACs* were detectable in roots, hypocotyls, cotyledons, euphylla, leaves and fruits from 10 dpa to the red ripe stage (Figure 2). The Class I members of *RPD3/HDA1* family, *SlHDA3, SlHDA1*, and *SlHDA4*, were all highly expressed in flowers but lowly expressed in later fruit stages. In addition, *SlHDA1* was also highly expressed in the red ripe stage (Figure 2). The transcript of the Class III member, *SlHDA5*, accumulated to a high level in flowers and 10 dpa, but decreased as fruit development and ripening (Figure 2). The *SlHDA6*, a member of Class IV, was highly expressed in the red ripe stage but lower expressed in cotyledons, flowers and fruits at 10, 20, and 30 dpa stages. Two members of Class II subfamily, *SlHDA7* and *SlHDA10*, shared the similar expression pattern and were highly expressed in cotyledons, euphylla and leaves (Figure 2). Unlike *SlHDA7* and *SlHDA10*, the other members of Class II subfamily, *SlHDA8* and *SlHDA9*, showed different expression patterns. *SlHDA8* was highly expressed in 10 dpa stage, while the *SlHDA9* transcript was accumulated in cotyledons and euphylla (Figure 2).

In comparison, the two members of *HD2* family, *SlHDT3* and *SlHDT1*, were highly expressed in roots and hypocotyls. In addition, transcripts of *SlHDT2* and *SlHDT1* were also accumulated in flowers and 10 dpa stage (Figure 2). Furthermore, the members of *SIR2* family, *SlSRT1* and *SlSRT2*, were highly expressed in cotyledons, flowers and 10 dpa stage (Figure 2).

### The subcellular localization of SlHDACs

WoLF PSORT was used to determine the predicted subcellular localizations of SlHDACs. Interestingly, SlHDACs were predicted to have various subcellular localizations including nuclei, peroxisomes, plasma membranes, cytoplasms, mitochondria and chloroplasts (Table 1).

**Figure 3:**
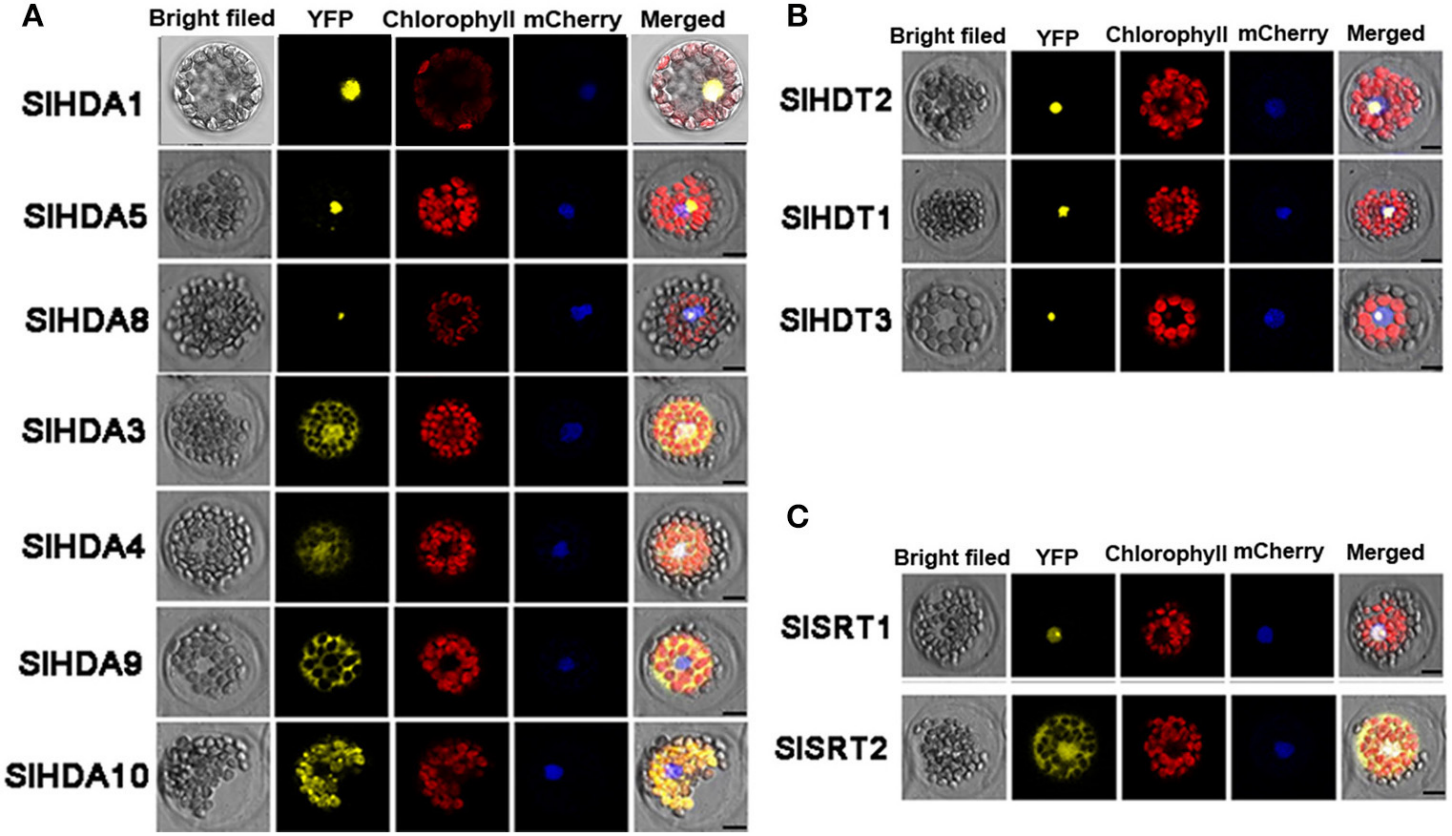
**Protoplast transient expression analysis using HDAC-YFP fusion constructs**. Subcellular location of RPD3/HDA1 **(A)**, HD2 **(B)**, and SRT2 **(C)** family HDACs was determined via *Arabidopsis* protoplast PEG transfection using HDAC-YFP fusion constructs. Red color indicated autofluorescence emitted by chloroplasts. The blue color indicates the nucleus using the mCherry as the nuclear marker. Bar represents 7.5 μm.

To further determine the subcellular localization of SlHDACs, the cDNA of each *SlHDAC* was fused with *Yellow Fluorescent Protein (YFP)* driven by the *Cauliflower mosaic virus* 35S promoter and transiently expressed in *Arabidopsis* protoplasts. As shown in Figure 3, SlHDACs of the three subfamilies showed different subcellular localization. The members of the RPD3/HDA1 subfamily displayed variable subcellular localizations. SlHDA1, SlHDA5, and SlHDA8 were localized in the nucleus, while SlHDA9 was only localized in the cytoplasm (Figure 3A). In contrast, SlHDA3 and SlHDA4 were localized in both the cytosol and nucleus, implying the possibility of shuttling between the nucleus and cytoplasm. Interestingly, SlHDA10 was localized in the chloroplast, which is consistent with the result of the *WoLF PSORT* programs analysis.

Consistent with the predicted localization using WoLF PSORT programs, SlSRT1 and all the three members of HD2 subfamily were localized in the nucleus (Table 1, Figures 3B,C). A recent study showed that the SRT2 in *Arabidopsis* was localized at the inner mitochondrial membrane (Konig et al., 2014). Nevertheless, SlSRT2, the homolog of *Arabidopsis* SRT2 in tomato, was localized in both the nucleus and cytosol (Figure 3C).

### Members of RPD3/HDA1 subfamily interact with MADS-box proteins

**Figure 4:**
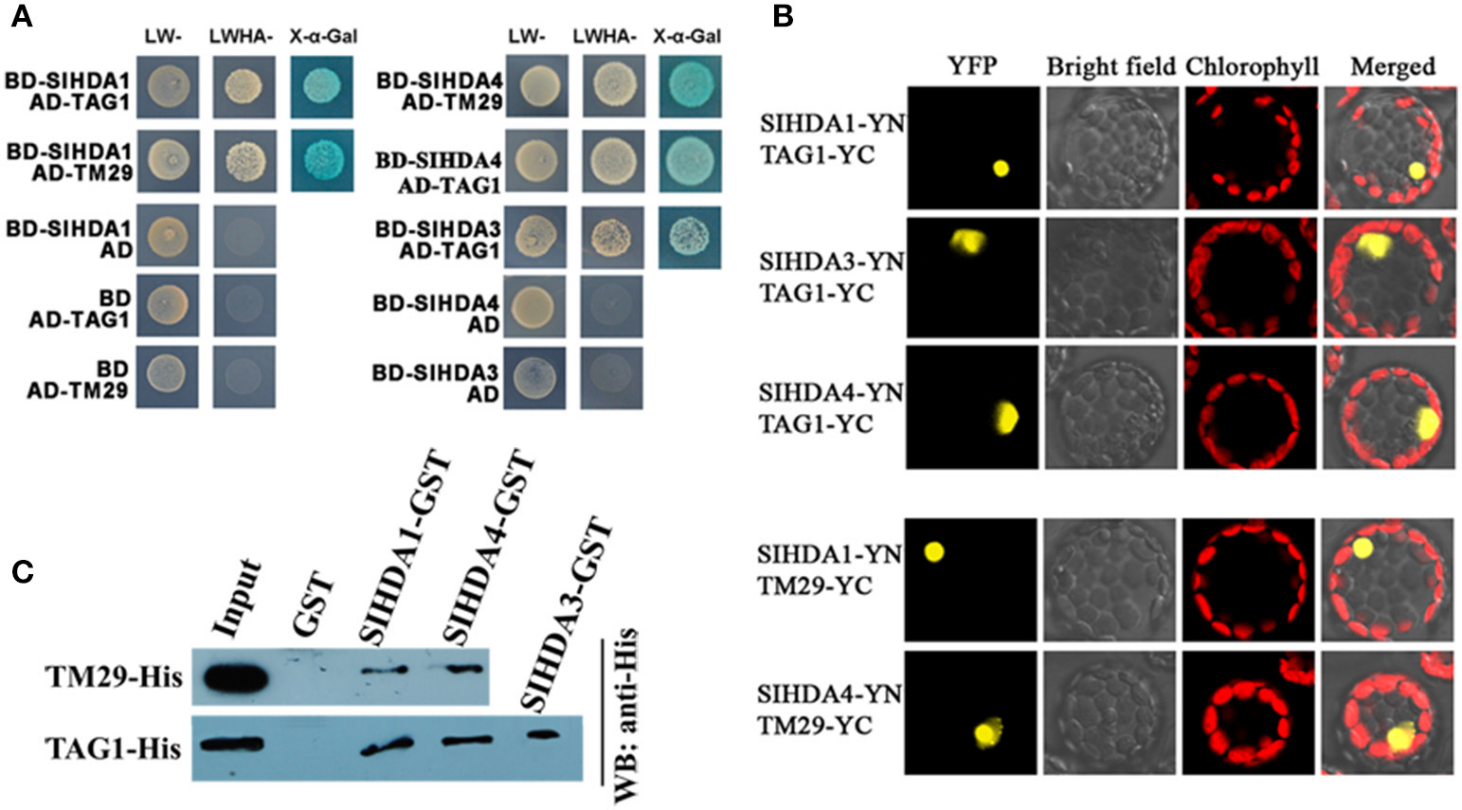
**SIHDACs interacted with MDAS-box proteins, TM29 and TAG1. (A)** SIHDACs interacted with TM29 and TAG1 in yeast two-hybrid assays. SlHDA1, SlHDA3, and SlHDA4 were cloned into pGADT7 vector, whereas TM29 and TAG1 were cloned into pGBKT7 vector, respectively. The plasmids were cotransformed into the yeast strain AH109. The transformants were grown on the selective minimal medium without Leu and Trp (LW-) or without Leu, Trp, Ade and His (LWHA-). SlHDACs interacted with TM29 and TAG1 in *Arabidopsis* protoplasts in BiFC assays. **(B)** SlHDA1/SlHDA2/SlHDA4 fused with the N terminus (YN) and TAG1/TM29 fused with the C terminus (YC) of YFP were cotransformed into protoplasts and then incubated in the 100 μmol.m-2.s-1 for 12 h. The fluorescence was determined using a confocal microscope. The YFP fluorescence was excited by a 514 nm laser and captured at 523–600 nm, and the chlorophyll autofluorescence was captured at 650–750 nm. Bar = 20 μm. SlHDACs interacted with TM29 and TAG1 in pull-down assays. **(C)** GST- SlHDAC1, GST- SlHDAC3, GST- SlHDAC4 or GST was incubated with either TM29-His or TAG1-His and GST affinity resin, and the bound proteins were then eluted from resin and probed with the anti-His antibody.

In *Arabidopsis*, two MADS-box proteins associated with flowering, AGAMOUS-like 15 (AGL15) and AGAMOUS-like 24 (AGL24), were shown to interact with SAP18, a subunit of the SIN3-HDAC complex involved in transcriptional repression (Hill et al., 2008; Liu et al., 2009). Furthermore, a recent study found that SlMADS1/LeMADS1 interacted with the N-terminal domain of mammalian HDAC5 *in vitro* (Gaffe et al., 2011). These data suggest that MADS transcription factors may be associated with a HDAC protein complex. The interactions between the members of RPD3/HDA1 family HDACs and the MADS-box proteins involved in fruit development were performed using yeast two-hybrid assays. Our results show that two MADS-box proteins, TM29 and TAG1, interacted with SlHDA1 and SlHDA4 (Figure 4A). In addition, SlHDA3, the homolog of *Arabidopsis* HDA6 in tomato, also interacted with TAG1 in yeast cells (Figure 4A). Interestingly, we also observed TM29 and TAG1 interacted with SlHDA6 and SlHDA7 in yeast cells (see Supplementary Figure 3).

The interaction of SlHDACs and these MADS-box proteins was further studied *in vivo* by the bimolecular fluorescence complementation (BiFC) and *in vitro* pull-down assays. For BiFC assays, SlHDACs and the MADS-box proteins were fused to either the N-terminal or C-terminal portion of YFP in the pSAT1 vector. The constructs were codelivered into *Arabidopsis* protoplasts and then incubated under light for 12 h. Strong YFP signal was observed in the nucleus when transiently coexpressing SlHDA1-YN/ SlHDA3-YN/ SlHDA4-YN/ with TAG1-YC (Figure 4B). Similarly, strong YFP signal was also observed in the nucleus when transiently coexpressing SlHDA1-YN/ SlHDA4-YN/ with TM29-YC (Figure 4B). As a control, no YFP signal was observed when these construct were cotransformed with the YN or NC empty vector (see Supplementary Figure 4).

For *in vitro* pull-down assays, purified TM29-His or TAG1-His recombinant protein was incubated with Glutathione S-Transferase (GST)-SIHDA1, GST-SIHDA3 or GST-SIHDA4 protein, respectively. As shown in Figure 4C, TM29-His was pulled down by GST-SIHDA1 and GST-SIHDA4. Similarly, TAG1-His was also pulled down by GST-SIHDA1, GST-SIHDA4 and GST-SIHDA3. These results indicate that TM29 is directly associated with SIHDA1 and SIHDA4, whereas TAG1 is directly associated with SIHDA1, SIHDA3 and SIHDA4.

## Discussion

In *Arabidopsis*, HDACs were found to be crucial players in all aspects of plant development including embryogenesis, abaxial/adaxial polarity determination, flowering, senescence, responses to day length and environmental stresses (Hollender and Liu, 2008; Ma et al., 2013; Liu et al., 2014). In comparison, relatively few HDACs were characterized in other plant species. In this study, 15 HDACs in tomato were identified using the bioinformatics analysis. The SlHDACs can be separated into three families: RPD3/HDA1, HD2 and SIR2 (Figure 1). Ten SlHDACs belong to the RPD3/HDA1 family and they all contain a typical deacetylase catalytic domain that is essential for the deacetylation activity (Figure 1B). In addition, the two members of the SIR2 family do not share sequence homology with other HDAC family members (Figure 1B). The three members of the HD2 family in tomato all contain a conserved pentapeptide (MEFWG) on the N-terminus of the protein, while the C-terminus has a variant domain (Additional file 1). A C_2_H_2_ zinc finger domain was identified on the C-terminus of the SlHDT1 and SlHDT2, which may participate in the protein-protein interaction (Figure 1). Compared with the previous report (Cigliano et al., 2013b), a new HDAC protein, SlHDA10, belonging to the RPD3/HDA1 family, was also found in the tomato (Table 1, Figure 1). The Conserved Domain search result clearly showed that SlHDA10 contains an Arginase HDAC domain (Accession NO. cl17011).

The development progress of tomato can be divided into several stages after the flower anthesis. Mature green (MG) stage, breaker stage (Br), turning stage (Tu) and the final red ripe stage (RR) are all the key development stages of tomato. MG stages can also be divided into different stages according to the gel production in locules, seed maturation and the external color (Teyssier et al., 2008). The fruit ripening is regulated by numerous genes which participate in different pathways influencing texture, color, pigment and aroma. Previous RNA-seq expression data demonstrate that *SlHDACs* may play roles in tomato fruit ripening, since *SlHDA1* and *SlHDA3* are highly expressed at B10 (10 days after breaking) and B fruit stages, respectively (Sato et al., 2012; Cigliano et al., 2013b). Consistent with this report, our data show that *SlHDA4, SlHDA8, SlHDA6*, and *SlHDT2* were all highly expressed in inflorescences to fruits development stages, indicating the potential role of *SlHDACs* in fruits development (Figure 2). *SlHDA3*, the close homolog of *AtHDA6* (Tanaka et al., 2008; Chen et al., 2010; Yu et al., 2011), was constitutively expressed in all stage of tomato development, especially in flowers (Figure 2). The two members of the SIR2 family show markedly different expression profiles, implying that they may be involved in different cellular processes (Figure 2). We also found that the expression profiles of some *SlHDACs* were not consistent with previous data based on RNA-seq analysis (Cigliano et al., 2013b). The discrepancy may be derived from the fact that plant materials at different developmental stages were used for the gene expression analysis. Interestingly, we found that the transcript of *SlHDA2* was not detected in all tissues and fruits of different development stages analyzed. Similarly, previous RNA-seq data also showed that the transcript level of *SlHDA2* is very low in tomato (Sato et al., 2012).

We found that the members of tomato RPD3/HDA1 family show a variety of subcellular localizations (Figure 3). SlHDA1, SlHDA5, and SlHDA8 were localized in the nucleus. In contrast, SlHDA3 and SlHDA4 were localized in both the nucleus and cytoplasm, suggesting the possibility of shuttling between the nucleus and cytoplasm. The shuttling of HDACs between the nucleus and the cytoplasm was previously reported in *Arabidopsis* and mammalian cells (Sengupta and Seto, 2004; Alinsug et al., 2009). In contrast, SlHDA9, the member Class II HDACs, was only localized in cytoplasm, implying that its substrates may be cytoplasmic proteins. Interestingly, SlHDA10 was localized in the chloroplast and the *SlHDA10* transcript was highly expressed in leaf tissues. It remains to be determined whether SlHDA10 deacetylates chloroplast proteins. Like their *Arabidopsis* homologs, the members of tomato HD2 subfamily were all localized in the nucleus (Zhou et al., 2004). Previous studies indicate that the *Arabidopsis* AtSRT2 is localized mitochondria (Konig et al., 2014). In contrast, we found that SlSRT2 was localized in both the nucleus and cytoplasm. The difference in subcellular localization suggests that different members of the same HDAC family may have distinctive functions in tomato.

To date, a number of MADS-box proteins attributing to fruit ripening of tomato have been identified (Vrebalov et al., 2002, 2009; Victoria et al., 2003; Giovannoni, 2007; Dong et al., 2013). In *Arabidopsis*, it was reported that the MADS-box proteins, AGL15 and AGL24, can interact with SAP18, a component of the SIN3/HDAC complex involved in transcriptional repression (Hill et al., 2008; Liu et al., 2009), suggesting that these MADS transcription factors may be associated with a HDAC protein complex. A previous study indicates that the tomato MADS protein SlMADS1/LeMADS1 interacts with the N-terminal domain of the mammalian HDAC5 *in vitro* (Gaffe et al., 2011). Our data show that SlHDA1 and SlHDA4 interacted TAG1 and TM29, the MADS-box proteins associated with tomato reproductive development (Figure 4). *TAG1* is a member of the AGAMOUS clade of MADS-box genes in tomato and it is expressed in flowers and ripening fruits. Suppression of the *TAG1* gene in tomato leads to a variety of floral defects and production of smaller amounts of pollen (Pan et al., 2010). In addition, TAG1 is necessary for the expression of both ethylene-dependent and independent genes during ripening (Klee and Giovannoni, 2011). On the other hand, *TM29*, a tomato *SEPALLATA* homolog, is highly expressed in the primordia of all four whorls of floral organs (Ampomah-Dwamena et al., 2002). Down-regulation of *TM29* via cosuppression or antisense techniques causes parthenocarpic fruits and aberrant flowers, as the petals and stamens are partially converted to a sepaloid identity (Ampomah-Dwamena et al., 2002). *SIHDAC1, SIHDAC4, TAG1*, and *TM29* all have peak expression at the flowering stage and all of them are expressed during fruit development (Ampomah-Dwamena et al., 2002; Pan et al., 2010) (See Supplementary Figure 5). The interaction of TAG1 and TM29 with SlHDA1 and SlHDA4 indicates that these MADA-box proteins may recruit HDACs to regulate gene expression in reproductive development in tomato.

## Conclusion

Fifteen SlHDACs identified from the genome of *Solanum lycopersicum* can be divided into RPD3/HDA1, SIR2, and HD2 families. Most *SlHDACs* were expressed in all tissues examined with different transcript abundance. SlHDA8, SlHDA1, SlHDA5, SlSRT1 and members of the HD2 family were localized to the nucleus, whereas SlHDA3 and SlHDA4 were localized in both the cytoplasm and nucleus. Furthermore, TAG1 and TM29 interacted with SlHDA1 and SlHDA4, indicating that these MADA-box proteins may recruit HDACs to regulate gene expression in reproductive development in tomato.

## Author contributions

Songguang Yang and Keqiang Wu conceived this project and designed all research. Linmao Zhao, Jingxia Lu, Pei-Ying Wu, and Jianxia Zhang performed the research. Songguang Yang and Keqiang Wu and Jingxia Lu and Linmao Zhao analyzed data. Songguang Yang and Keqiang Wu wrote the article.

### Conflict of interest statement

The authors declare that the research was conducted in the absence of any commercial or financial relationships that could be construed as a potential conflict of interest.

## Abbreviations

bp: base pair
BiFC: bimolecular fluorescence complementation
cDNA: complementary DNA
dpa: days post anthesis
GST: glutathione S-transferase
HATs: histone acetyltransferases
HDACs: histone deacetylases
MADS-box: Minichromosome Maintenance 1, Agamous, Deficiens and human serum response factor SRF
MG: mature green
PCR: polymerase chain reaction
RR: red ripe
*SEP*: *SEPELATTA*
*TAG1*: *TOMATO AGAMOUS11*
*TAGL11*: *TOMATO AGAMOUS-LIKE1*
*TM4*: *TOMATO MADS BOX4*
*TM29*: *TOMATO MADS BOX29*
YFP: yellow fluorescence proteins.
