# Supplementary material for: Identification and characterization of histone deacetylases in tomato (Solanum lycopersicum)
Source: Front Plant Sci. 2015 Jan 6;5:760. doi: 10.3389/fpls.2014.00760 (PMC4285013; doi:10.3389/fpls.2014.00760)
Supplement: Supplementary file 1 [file Presentation1.PDF]

Supplementary Material

Identification and Characterization of Histone Deacetylases in Tomato (Solanum Lycopersicum)

Linmao Zhao[1, 2 a], Jingxia Lu[1, 2 a], Jianxia Zhang[1, 3], Pei-Ying Wu[3], Songguang Yang[1*] and Keqiang Wu[3*]

[1] Key Laboratory of Plant Resources Conservation and Sustainable Utilization, South China Botanical Garden, Chinese Academy of Sciences, Guangzhou, China

[2] University of Chinese Academy of Sciences, Beijing, China

[3] Institute of Plant Biology, National Taiwan University, Taipei 106, Taiwan

[a] These authors contributed equally to this work.

[*] Correspondence: yangsongguang@scbg.ac.cn; kewu@ntu.edu.tw

1. Suplementary Tables

Supplementary Table 1 Primers used in this study

Primer name	Forward	Reverse
qRT-PCR primers		
SlHDA1	TAAGCCTGGTGCTGTGGTT	CAGCAACGAGCAACATTACG
SlHDA3	AAGCCGCACCGTATCAGAAT	GGTGAAACGGTAGCAAGGAA
SlHDA2	CGGTGATGTAGGAAGCGTAT	GTTCTGAGTATCTGGGGTTATTCT
SlHDA4	CGCCGTAGCCGTATTCTC	TCATCTTTAGTTGCCTCCACA
SlHDA5	GGAGGACAGGTGGAAGGC	TCAGGGTGAAGAAAAGCAAC
SlHDA9	ATGAGGTTGGTTCTATGGGTG	CGTCACAGCAGCCAAGGGGGTCAC
SlHDA8	GCCTTCTACGAGGACCCA	AAACTGTTCGCATTGCTGTAT
SlHDA7	GCTTTAGATGGTGGCTGTAGG	ATAGTTCCACCCTGTGAATGAG
SlHDA6	GGGTCATAATCAGGAATCACAT	CCTTCTCAAGCCCTGAAACAT
SlHDA10	GGGCAATGTTGGGATGAC	GGCAATGATGCTTCTGGTAG
SlSRT1	TGAAAACGGCAGCACGAA	GCTCCCGTCAATACAACAAG
SlSRT2	ATTTTGGGGTGCGGAGGT	GCTTCTTGCCATCAATAGTTACAG
SlHDT2	CAACAAGTCAGGTGGTAAGAAAGG	CTTCCTTGCTTGCCCTTG

SlHDT3	GATACAAGGCAACTAACCCG	TCTTTAGTAGGCTCCACAATCC
SlActin	CCAAGGCCAACAGAGAGAAG	GAAAGCACAGCCTGGATAGC
primers used for cloning gene		
SlHDA1	ATGGATGTTGGAGGAAACTCCTT	TTAGGAGATAATATCAGTTGGTT
SlHDA3	ATGGACTCCTCCACCGTAGACGG	CTAGGGATGATCATCAACCATGT
SlHDA2	ATGAGGTCCAAGGACAAAATCTC	TTAGGCATCATCAGTGTGGTTAT
SlHDA4	ATGTCATCCGCTGCCTCTTCCTC	CTAGCTCTTCATATCTATCAGGG
SlHDA5	ATGGATTCCGGTGAACGACGGCG	TTACAATGCTCTGCTAGCTGCTT
SlHDA9	ATGATCTTAGTGCAAAAATGTGT	TCAACAAGAAAAATTACAAGAGG
SlHDA8	ATGCAGACATTCCAAGAGTCGTT	TCAAAAGGAATGTATGTGCTTCA
SlHDA7	ATGGCTTCTTCAGCATCACAATC	CTAAGCATCTCTAAGAAAAGGTA
SlHDA6	ATGAAGTCTGTATCCTCTCTACC	TTACAATGGTGTGGCAGTAGCAT
SlHDA10	ATGTCTCTGGGTTATGCTGAAAA	TCACCTTACATTAATTACAGTTC
SlSRT1	ATGTCCCTGCGACTTTGTTGCAG	CTAGGGAGCAGGGATACTTAATG
SlSRT2	ATGGAATTTTGGGGTGCGGAGGT	TTACTTTCCAGCGCTGTGCTTAG
SlHDT2	ATGGAGTTTTGGGGTGTGACATC	CTACTTCCTTCCTTGCTTGCCCT
SlHDT3	ATGGAGTTTTGGGGTGCTGAGGT	TTACTTTCCAGCACTGTGTTTAG
SlHDA1-BiFC	TCTCGAGCTCAAGCTTCGATGGATGTTGGAGGAAACTCCTT	GCAGAATTCGAAGCTTGTTAGGAGATAATATCAGTTGGTT
SlHDA2-BiFC	TCTCGAGCTCAAGCTTCGATGGACTCCTCCACCGTAGACGG	GCAGAATTCGAAGCTTGGGGATGATCATCAACCATGTCAT
SlHDA4-BiFC	TCTCGAGCTCAAGCTTCGATGAGGTCCAAGGACAAAATCTC	GCAGAATTCGAAGCTTGTTAGGCATCATCAGTGTGGTTAT
TAG1-BiFC	TCTCGAGCTCAAGCTTCGATGGACTTCCAAAGTGATCTAAC	GCAGAATTCGAAGCTTGTTAGACTAGTTGAATAGGGGGTT
TM29-BiFC	TCTCGAGCTCAAGCTTCGATGGGTAGAGGAAGAGTTGAGCT	GCAGAATTCGAAGCTTGTCACAGCATCCAACCAGGTATCA
SlHDA1-BD	CATGGAGGCCGAATTCATGGATGTTGGAGGAAACTCCTT	GGATCCCCGGGAATTCTTAGGAGATAATATCAGTTGGTT
SlHDA2-BD	CATGGAGGCCGAATTCATGGACTCCTCCACCGTAGACGG	GGATCCCCGGGAATTCGGGATGATCATCAACCATGTCAT
SlHDA4-BD	CATGGAGGCCGAATTCATGAGGTCCAAGGACAAAATCTC	GGATCCCCGGGAATTCTTAGGCATCATCAGTGTGGTTAT
TAG1-AD	GGAGGCCAGTGAATTCATGGACTTCCAAAGTGATCTAAC	CACCCGGGTGGAATTCTTAGACTAGTTGAATAGGGGGTT

TM29-AD	GGAGGCCAGTGAATTCATGGGTAGAGGAA GAGTTGAGCT	CACCCGGGTGGAATTCTCACAGCATCCAAC CAGGTATCA
SlHDA1-GST	GTGGATCCCCGAATTCCATGGATGTTGGAG GAAACTCCTT	AGTCGACCCGGGAATTCGGAGATAATATCA GTTGGTTGAT
SlHDA3-GST	GTGGATCCCCGAATTCCATGGACTCCTCCA CCGTAGACGG	AGTCGACCCGGGAATTCGGGATGATCATCA ACCATGTCAT
SlHDA4-GST	GTGGATCCCCGAATTCCATGAGGTCCAAG GACAAAATCTC	AGTCGACCCGGGAATTCGGCATCATCAGTG TGGTTATCGT
TM29-His	AGGCCATGGCTGATATCATGGGTAGAGGA AGAGTTGAGCT	CGACGGAGCTCGAATTCCAGCATCCAACCA GGTATCATAC
TAG1-His	AGGCCATGGCTGATATCATGGACTTCCAAA GTGATCTAAC	CGACGGAGCTCGAATTCGACTAGTTGAATA GGGGGTTGGT

2. Suplementary Figures

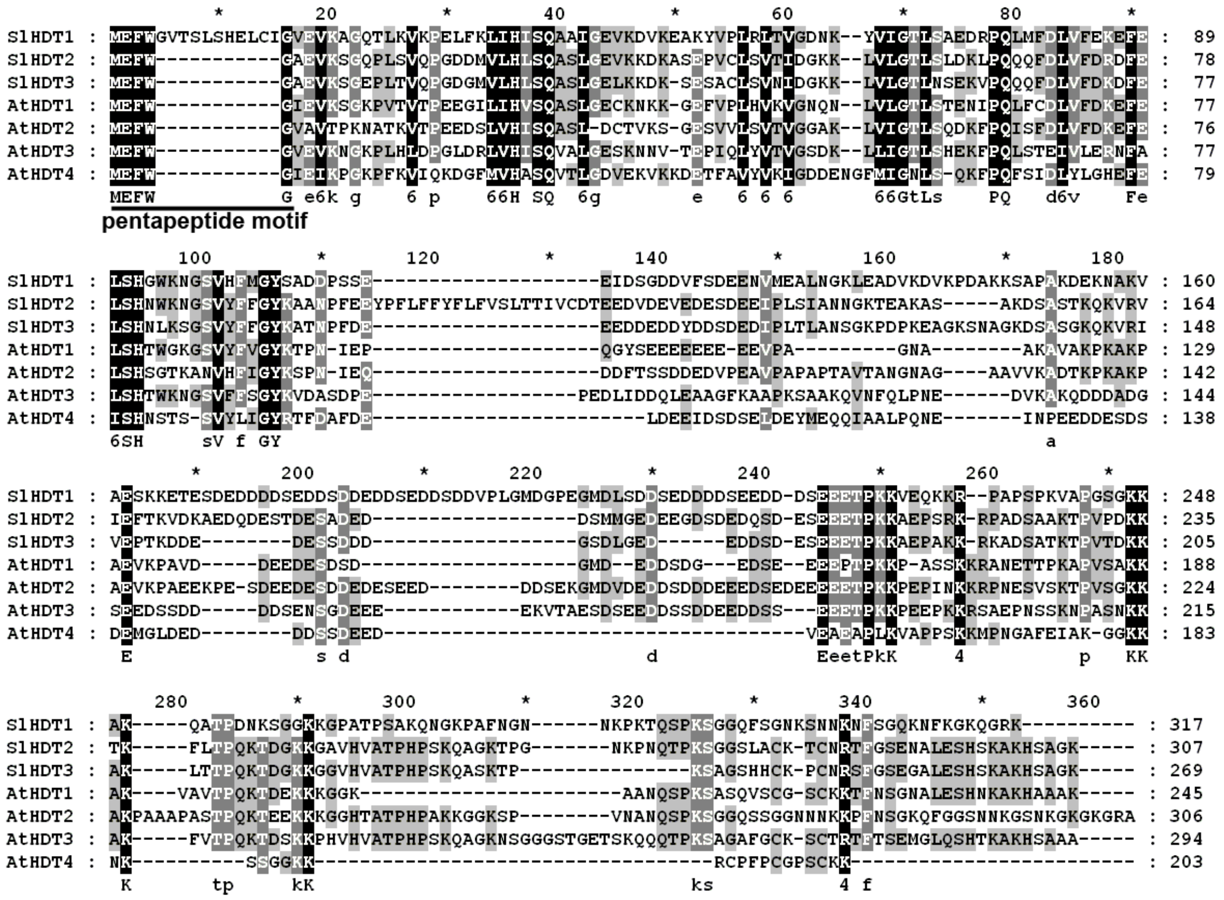

Supplementary Figure 1. Sequence features of HDT-type HDACs in tomato and Arabidopsis. The pentapeptide motif is shown.

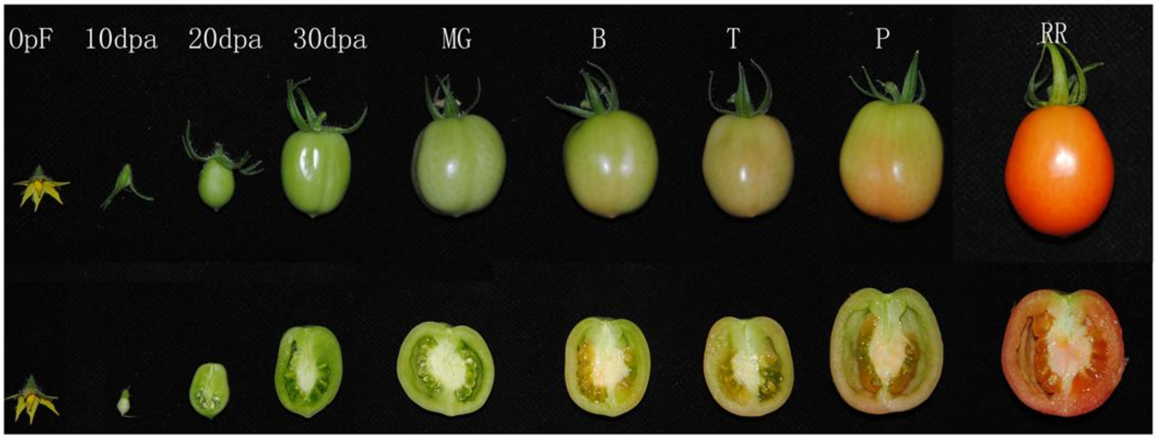

Supplementary Figure 2. The flower and fruit samples are as follows: open flower (OpF), fruits at 10 days post anthesis (30 dpa), 20 and 30 dpa, mature green (MG), breaker stage (B), turning stage (T) pink stage (P) and red ripe stage (RR).

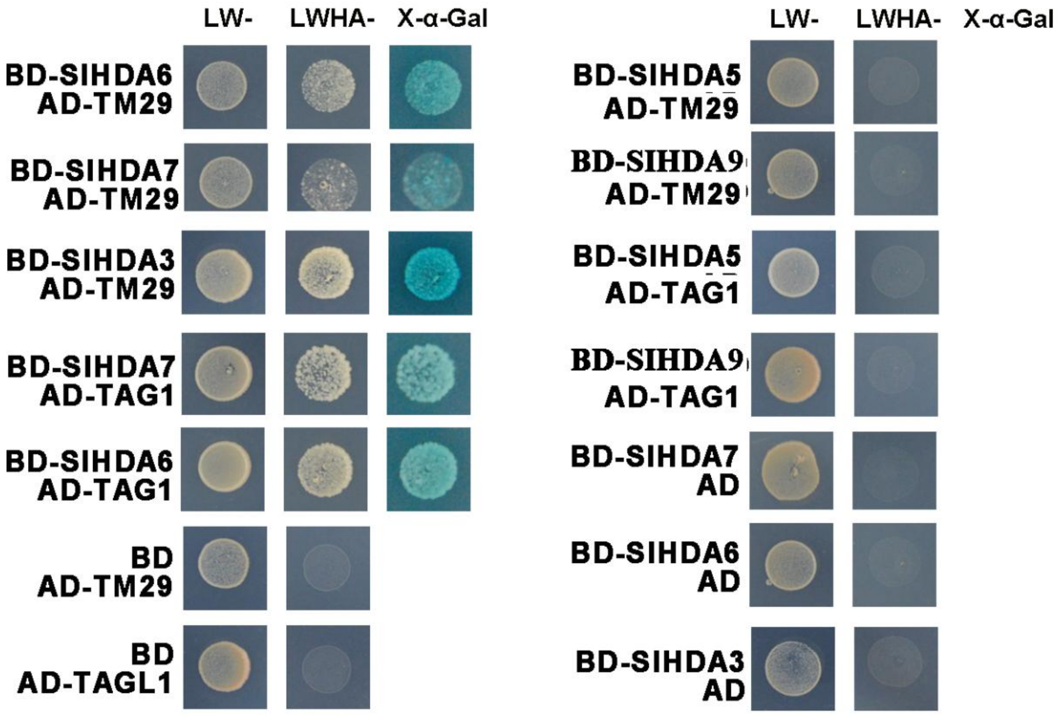

Supplementary Figure 3. Yeast two-hybrid analysis of SlHDACs interated with TM29 and TAG1 proteins.

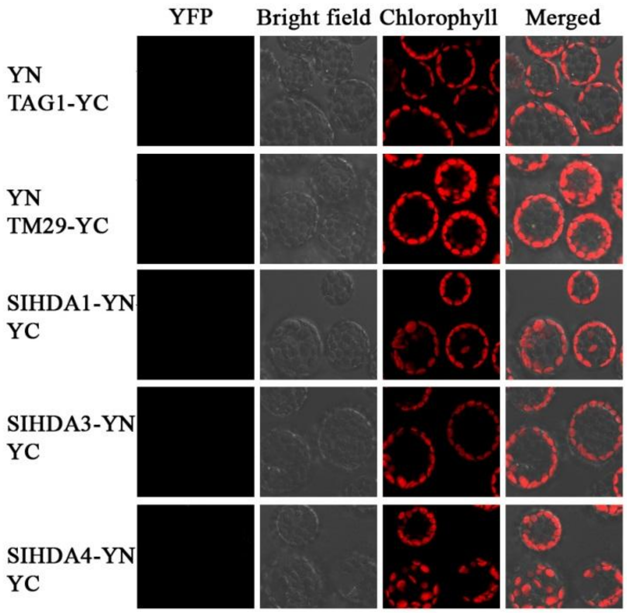

Supplementary Figure 4. The negative controls of BiFC assays.

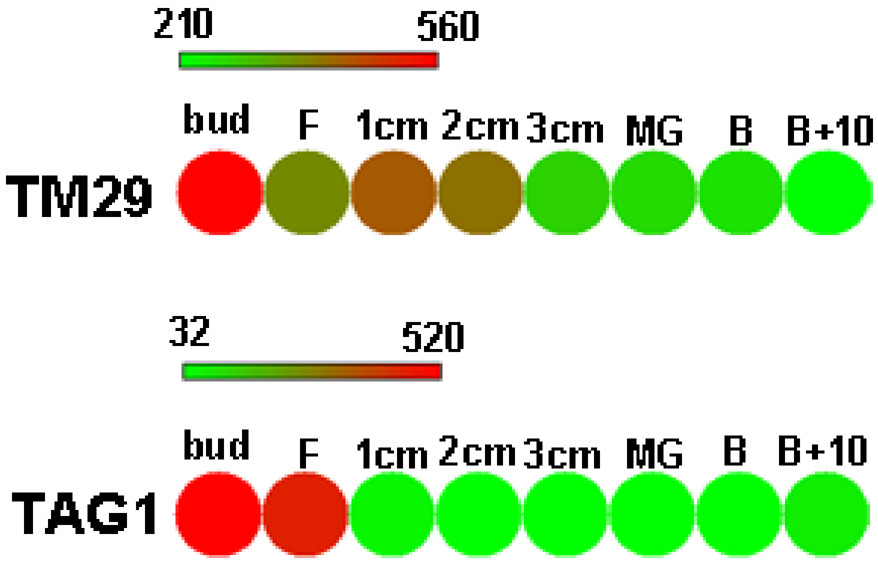

Supplementary Figure 5. Expression profiles of TM29 and TAG1. Heatmap of RNA-seq expression data from bud, flower (F), 1cm_fruit (1cm), 2cm_fruit (2cm), 3cm_fruit (3cm), mature green fruit (MG), berry at breaker stage (B) and berry ten days after breaker stage (B+10). The expression values are measured as reads per kliobase of exon model per million mapped reads (RPKM).
