# Supplementary material for: Identification and characterization of histone deacetylases in tomato (Solanum lycopersicum)
Source: Front Plant Sci. 2015 Jan 6;5:760. doi: 10.3389/fpls.2014.00760 (PMC4285013; doi:10.3389/fpls.2014.00760)
Supplement: Supplementary file 2 [file DataSheet1.DOCX]

***Supplementary Material***

**Molecular Characterization of Histone Deacetylases in Tomato (*Solanum Lycopersicum*)**

Linmao Zhao^1, 2 a^, Jingxia Lu^1, 2 a^, Jianxia Zhang^1, 3^, Pei-Ying Wu^3^, Songguang Yang^1*^ and Keqiang Wu^3*^

^1^ Key Laboratory of Plant Resources Conservation and Sustainable Utilization, South China Botanical Garden, Chinese Academy of Sciences, Guangzhou, China

^2^ University of Chinese Academy of Sciences, Beijing, China

^3^ Institute of Plant Biology, National Taiwan University, Taipei 106, Taiwan

^a^ These authors contributed equally to this work.

^*^ Correspondence: [yangsongguang@scbg.ac.cn](http://mail.cstnet.cn/coremail/XJS/pab/view.jsp?sid=BAPKDVOOuhHHldbYAuOOHRnGbulemDwj&totalCount=12&view_no=8&puid=47&gid=0); [kewu@ntu.edu.tw](mailto:kewu@ntu.edu.tw)

## Suplementary Tables

Supplementary Table 1 Primers used in this study

| Primer name | Forward | | | Reverse |
| --- | --- | --- | --- | --- |
| qRT-PCR primers | | | | |
| *SlHDA1* | | | TAAGCCTGGTGCTGTGGTT | CAGCAACGAGCAACATTACG |
| *SlHDA3* | | | AAGCCGCACCGTATCAGAAT | GGTGAAACGGTAGCAAGGAA |
| *SlHDA2* | | | CGGTGATGTAGGAAGCGTAT | GTTCTGAGTATCTGGGGTTATTCT |
| *SlHDA4* | | | CGCCGTAGCCGTATTCTC | TCATCTTTAGTTGCCTCCACA |
| *SlHDA5* | | | GGAGGACAGGTGGAAGGC | TCAGGGTGAAGAAAAGCAAC |
| *SlHDA9* | | | ATGAGGTTGGTTCTATGGGTG | CGTCACAGCAGCCAAGGGGGTCAC |
| *SlHDA8* | | | GCCTTCTACGAGGACCCA | AAACTGTTCGCATTGCTGTAT |
| *SlHDA7* | | | GCTTTAGATGGTGGCTGTAGG | ATAGTTCCACCCTGTGAATGAG |
| *SlHDA6* | | | GGGTCATAATCAGGAATCACAT | CCTTCTCAAGCCCTGAAACAT |
| *SlHDA10* | | | GGGCAATGTTGGGATGAC | GGCAATGATGCTTCTGGTAG |
| *SlSRT1* | | | TGAAAACGGCAGCACGAA | GCTCCCGTCAATACAACAAG |
| *SlSRT2* | | | ATTTTGGGGTGCGGAGGT | GCTTCTTGCCATCAATAGTTACAG |
| *SlHDT2* | | | CAACAAGTCAGGTGGTAAGAAAGG | CTTCCTTGCTTGCCCTTG |
| *SlHDT3* | | | GATACAAGGCAACTAACCCG | TCTTTAGTAGGCTCCACAATCC |
| *SlActin* | | | CCAAGGCCAACAGAGAGAAG | GAAAGCACAGCCTGGATAGC |
| primers used for cloning gene | | | | |
| *SlHDA1* | | ATGGATGTTGGAGGAAACTCCTT | | TTAGGAGATAATATCAGTTGGTT |
| *SlHDA3* | | ATGGACTCCTCCACCGTAGACGG | | CTAGGGATGATCATCAACCATGT |
| *SlHDA2* | | ATGAGGTCCAAGGACAAAATCTC | | TTAGGCATCATCAGTGTGGTTAT |
| *SlHDA4* | | ATGTCATCCGCTGCCTCTTCCTC | | CTAGCTCTTCATATCTATCAGGG |
| *SlHDA5* | | ATGGATTCCGGTGAACGACGGCG | | TTACAATGCTCTGCTAGCTGCTT |
| *SlHDA9* | | ATGATCTTAGTGCAAAAATGTGT | | TCAACAAGAAAAATTACAAGAGG |
| *SlHDA8* | | ATGCAGACATTCCAAGAGTCGTT | | TCAAAAGGAATGTATGTGCTTCA |
| *SlHDA7* | | ATGGCTTCTTCAGCATCACAATC | | CTAAGCATCTCTAAGAAAAGGTA |
| *SlHDA6* | | ATGAAGTCTGTATCCTCTCTACC | | TTACAATGGTGTGGCAGTAGCAT |
| *SlHDA10* | | ATGTCTCTGGGTTATGCTGAAAA | | TCACCTTACATTAATTACAGTTC |
| *SlSRT1* | | ATGTCCCTGCGACTTTGTTGCAG | | CTAGGGAGCAGGGATACTTAATG |
| *SlSRT2* | | ATGGAATTTTGGGGTGCGGAGGT | | TTACTTTCCAGCGCTGTGCTTAG |
| *SlHDT2* | | ATGGAGTTTTGGGGTGTGACATC | | CTACTTCCTTCCTTGCTTGCCCT |
| *SlHDT3* | | ATGGAGTTTTGGGGTGCTGAGGT | | TTACTTTCCAGCACTGTGTTTAG |
| *SlHDA1-*BiFC | | TCTCGAGCTCAAGCTTCGATGGATGTTGGAGGAAACTCCTT | | GCAGAATTCGAAGCTTGTTAGGAGATAATATCAGTTGGTT |
| *SlHDA2-*BiFC | | TCTCGAGCTCAAGCTTCGATGGACTCCTCCACCGTAGACGG | | GCAGAATTCGAAGCTTGGGGATGATCATCAACCATGTCAT |
| *SlHDA4-*BiFC | | TCTCGAGCTCAAGCTTCGATGAGGTCCAAGGACAAAATCTC | | GCAGAATTCGAAGCTTGTTAGGCATCATCAGTGTGGTTAT |
| *TAG1-*BiFC | | TCTCGAGCTCAAGCTTCGATGGACTTCCAAAGTGATCTAAC | | GCAGAATTCGAAGCTTGTTAGACTAGTTGAATAGGGGGTT |
| *TM29-*BiFC | | TCTCGAGCTCAAGCTTCGATGGGTAGAGGAAGAGTTGAGCT | | GCAGAATTCGAAGCTTGTCACAGCATCCAACCAGGTATCA |
| *SlHDA1-*BD | | CATGGAGGCCGAATTCATGGATGTTGGAGGAAACTCCTT | | GGATCCCCGGGAATTCTTAGGAGATAATATCAGTTGGTT |
| *SlHDA2-*BD | | CATGGAGGCCGAATTCATGGACTCCTCCACCGTAGACGG | | GGATCCCCGGGAATTCGGGATGATCATCAACCATGTCAT |
| *SlHDA4-*BD | | CATGGAGGCCGAATTCATGAGGTCCAAGGACAAAATCTC | | GGATCCCCGGGAATTCTTAGGCATCATCAGTGTGGTTAT |
| *TAG1-*AD | | GGAGGCCAGTGAATTCATGGACTTCCAAAGTGATCTAAC | | CACCCGGGTGGAATTCTTAGACTAGTTGAATAGGGGGTT |
| *TM29-*AD | | GGAGGCCAGTGAATTCATGGGTAGAGGAAGAGTTGAGCT | | CACCCGGGTGGAATTCTCACAGCATCCAACCAGGTATCA |
| *TM29-AD*  *SIHDA1-GST*  *SIHDA3-GST*  *SIHDA4-GST*  *TM29-His*  *TAG1-His* | | GGAGGCCAGTGAATTCATGGGTAGAGGAA  GAGTTGAGCT  GTGGATCCCCGAATTCCATGGATGTTGGAGGAAACTCCTT  GTGGATCCCCGAATTCCATGGACTCCTCCACCGTAGACGG  GTGGATCCCCGAATTCCATGAGGTCCAAGGACAAAATCTC  AGGCCATGGCTGATATCATGGGTAGAGGAAGAGTTGAGCT  AGGCCATGGCTGATATCATGGACTTCCAAAGTGATCTAAC | | CACCCGGGTGGAATTCTCACAGCATCCAACCAGGTATCA  AGTCGACCCGGGAATTCGGAGATAATATCAGTTGGTTGAT  AGTCGACCCGGGAATTCGGGATGATCATCAACCATGTCAT  AGTCGACCCGGGAATTCGGCATCATCAGTGTGGTTATCGT  CGACGGAGCTCGAATTCCAGCATCCAACCAGGTATCATAC  CGACGGAGCTCGAATTCGACTAGTTGAATAGGGGGTTGGT |
